# Role of Alexithymia, Anxiety, and Depression in Predicting Self-Efficacy in Academic Students

**DOI:** 10.1155/2017/5798372

**Published:** 2017-01-05

**Authors:** Mahbobeh Faramarzi, Soraya Khafri

**Affiliations:** ^1^Social Determinants of Health Research Center, Health Research Institute, Babol University of Medical Sciences, Babol, Iran; ^2^Biostatistics & Epidemiology Department, Babol University of Medical Science, Babol, Iran

## Abstract

*Objective*. Little research is available on the predictive factors of self-efficacy in college students. The aim of the present study is to examine the role of alexithymia, anxiety, and depression in predicting self-efficacy in academic students.* Design*. In a cross-sectional study, a total of 133 students at Babol University of Medical Sciences (Medicine, Dentistry, and Paramedicine) participated in the study between 2014 and 2015. All participants completed the Toronto Alexithymia Scale (TAS-20), College Academic Self-Efficacy Scale (CASES), and 14 items on anxiety and depression derived from the 28 items of the General Health Questionnaire (28-GHQ).* Results*. Pearson correlation coefficients revealed negative significant relationships between alexithymia and the three subscales with student self-efficacy. There was no significant correlation between anxiety/depression symptoms and student self-efficacy. A backward multiple regression analysis revealed that alexithymia was a negative significant predictor of self-efficacy in academic students (*B* = −0.512, *P* < 0.001). The prevalence of alexithymia was 21.8% in students. Multiple backward logistic analysis regression revealed that number of passed semesters, gender, mother's education, father's education, and doctoral level did not accurately predict alexithymia in college students.* Conclusion*. As alexithymia is prevalent in college students and affects self-efficacy and academic functioning, we suggest it should be routinely evaluated by mental physicians at universities.

## 1. Introduction

Alexithymia is an inability to understand, process, or describe feelings. It refers to a personality trait associated with difficulties with identifying or describing feelings, mental imaging and fantasy, and external cognitive orientation [1]. Research has revealed that alexithymia is associated with psychiatric disorders, such as psychosomatic disorders [2–4], depression [5], and anxiety [6]. Some studies have reported a positive relationship between alexithymia and pain disorders [7, 8]. Alexithymia influences academic achievement of college students, some report moderately so [9]. Sepahvand et al. (2015) demonstrated a positive relationship between alexithymia and test anxiety in students [10].

Various psychiatric disorders are more frequent in college students than in nonstudent populations of a similar age group, owing to multiple stressors, including academic overload, inadequate time, and final examinations [11, 12]. Evidence has shown a marked increase in psychiatric disorders, including depression and anxiety in college students over the last decade [13]. Many environmental and personal factors may prevent psychiatric symptoms in academic students. Personal characteristics such as self-esteem, social acceptance, self-worth, and hopefulness affect mental health in college students [14, 15]. Another factor related to mental health in academic students is self-efficacy [16]. Self-efficacy refers to how individuals judge their ability to achieve their desired performance levels [17]. Research indicates that self-efficacy is strongly correlated with motivation, learning, and achievement [18, 19]. In a systematic review, self-efficacy correlated most strongly with mental health, followed by academic success [20].

Previous studies indicate a high degree of alexithymia in medical students [8, 21] and the influence of alexithymia on mental health [22]. Low self-efficacy was associated with depression and anxiety [23]. There are numerous gaps in the academic mental health literature: whether low self-efficacy is associated with alexithymia, whether alexithymia is related to depression and anxiety in academic students, and which factors impact alexithymia in academic students. The current study addresses the existing gap in the literature by testing the role of alexithymia in anxiety, depression, and self-efficacy. To the authors' knowledge, this is the first study to examine the impact of three factors on self-efficacy in academic students. The findings could offer a new way of understanding the self-efficacy of academic students. The aims of the study are to explore the following:

1. whether alexithymia is associated with self-efficacy,
2. whether anxiety and depression are associated with self-efficacy, and
3. how these factors relate to alexithymia in the students.

## 2. Materials and Method

### 2.1. Participants

The sample for this cross-sectional study was selected from a study (ID: 2098; 2014), conducted by the Social Determinants of Health Research Center (SDHRC) at the University of Babol of Medical Sciences. The Medical Ethics Committee of the university approved the study. The protocol assessed 133 Medical Sciences students between 2014 and 2015. 200 students agreed to participate; of these, 133 subjects completed the questionnaires. The sample included students from three colleges (Medicine, Dentistry, and Paramedicine) registered for the first and second semesters. Three questionnaires were chosen based on the aim of the study: the Toronto Alexithymia Scale (TAS-20), the College Academic Self-Efficacy Scale (CASES), and 14 items on anxiety and depression derived from the 28 items of the General Health Questionnaire (28-GHQ).

### 2.2. Instruments

#### 2.2.1. Toronto Alexithymia Scale (TAS-20)

Alexithymia was evaluated using the self-report 20-item TAS-20. It contains 20 items and three subscales that cover difficulty identifying feelings, difficulty describing feelings, and externally oriented thinking. The items require responses on a five-point scale (1 through 5). Total TAS-20 thus ranges from 20 to 100 [24]. We used cutoff scores of ≤60 for nonalexithymia and ≥61 for alexithymia [25]. A validated Persian version of the TAS-20 was used in this study [26].

#### 2.2.2. College Academic Self-Efficacy Scale (CASES)

This scale was developed by Owen and Froman (1988). The alpha coefficient validity of this scale was calculated at 0.90. It consists of 33 items ranging from 1 (very little) to 5 (a lot) [27]. Persian valid CASES were used in this study [28].

#### 2.2.3. General Health Questionnaire (GHQ-28)

This contains 28 items and four subscales: somatic symptoms, anxiety symptoms, impairments in social functioning, and depression symptoms. In this study, two of the subscales, depression symptoms and anxiety symptoms, were used, which encompass 14 items [29]. The scale has been validated in the Iranian population [30].

### 2.3. Data Analysis

Pearson correlation analyses were performed using the scores from three instruments to verify associations between alexithymia, depression, and anxiety symptoms and self-efficacy. A multiple backward analyses regression was conducted to examine the specific contribution of alexithymia and gender to predicting self-efficacy in students. Also, we used multiple backward logistic analysis regression to explain the relationship between alexithymia and demographic characteristics of the population.

## 3. Results

Table 1 presents the demographic characteristics of the sample study. 62.4% of the students were women. The mean age of men and women was 20.37 ± 3.16 and 21.46 ± 5.65, respectively.

We also examined correlations between depression symptoms, anxiety symptoms, and alexithymia with self-efficacy in students. As described in Table 2, of the three subscales, only alexithymia was negatively related to self-efficacy in students. There was no significant correlation between anxiety symptoms and depression symptoms with self-efficacy of the students.

A backward multiple analyses regression was performed to examine the specific contribution of alexithymia and gender to predicting self-efficacy in students. The results of these analyses are presented in Table 3. Gender was not a significant predictor of self-efficacy. Alexithymia emerged as a negative significant predictor of self-efficacy in academic students (*B* = −0.512, *P* < 0.001).

21.8% of the students were alexithymic: of these, 48.1% were men and 51.9% women. We used multiple backward logistic analysis regression to explain the relationship between alexithymia and demographic characteristics of the population. As shown in Table 4, doctoral level predicted alexithymia in Step 1 (*B* = 0.395, *P* < 0.05). However, when the other four variables (number of semesters passed, gender, mother's education, and father's education) were included in the model (Step 5), doctoral level no longer accurately predicted alexithymia (*B* = 0.519, *P* > 0.05).

## 4. Discussion

The findings of the present study confirmed hypothesis 1: alexithymia is as a significant negative predictor of self-efficacy in academic students. According to further analysis, each of three alexithymia factors was negatively correlated with self-efficacy. Consistent with our study, Chung et al. (2013) reported that self-efficacy was significantly and negatively correlated with alexithymia [31]. Also, Pecukonis (2009) reported that women with chronic back pain were significantly more alexithymic and had lower self-efficacy, compared with control subjects [32].

How we can explain the negative predictor effect of alexithymia on self-efficacy in academic students? First, the ability to regulate feelings is self-efficacious. People, who recognize their feelings, understand their implicit content, and express their emotional states are better able to face the challenges of life [33]. Second, alexithymia is associated with impaired interpersonal skills and fewer close relationships [34]. On the other hand, research confirms that communication skills are important predictors of social self-efficacy [35]. Third, emotion regulation difficulty is associated with lower self-efficacy in college students, making it more likely that those with behavior disorders will avoid problems [36].

The results disconfirm hypothesis 2 that anxiety and depression are related to self-efficacy. In contrast to the present study, previous work suggests that self-efficacy is negatively related to depression and anxiety [23, 37]. Why did our results not confirm those of previous studies? Possibly, this was due to the nature of both the population and of the self-efficacy measure. First, previous studies used physical self-efficacy instruments to identify the relationship between self-efficacy and depression or anxiety [37]. Also, the sample groups were high school students [37], or patients with somatic disorder [23]; college students were not included. In our study, no relationship was noted between alexithymia and depression or anxiety, in stark contrast to previous studies. Michael Bagby et al. (1986) reported results from the TAS-20 and the Beck Depression Inventory (BDI) in university students [38]. Parker et al. (1991) showed that, although there was an association between alexithymia and depression in both nonclinical and clinical samples, the results of the factor analysis of the combined items from the TAS and BDI provided evidence that alexithymia was a construct distinct from depression [39]. The discrepancy in results might be attributable to nature of the depression and anxiety measure. We used the depression and anxiety subscales of the GHQ-28 to report depression and anxiety symptoms, whereas previous studies have used the BDI.

According to our results, alexithymia is prevalent in college students (21.8%). Little is known about alexithymia in academic students. In one study, the prevalence of alexithymia was reported as 23% and 17.1% in the normal and the student group, respectively [40]. The prevalence of alexithymia in France was reported at 20.7% [41]. Two other studies reported that the prevalence of alexithymia was higher in medical sciences students [8, 21]. Our results also failed to confirm hypothesis 3. We showed that the number of semesters passed, gender, mother's education, father's education, and level of education did not predict alexithymia. One study reported that female students had higher rates of alexithymia than males did [42]. Another study reported that men had higher scores on alexithymia than women did [43]. Comparative population studies in other countries are needed to determine whether there are any differences in the prevalence of alexithymia and related factors between academic students with different cultures. Some argue that alexithymia is better defined as a state or trait. Various findings support the view that this construct represents a stable personality trait [2, 44]. Other evidence reported that alexithymia was a state-dependent personality construct and that TAS-20 scores changed after psychotherapy [45]. Luminet et al. [46] evaluated both the absolute stability and the relative stability of alexithymia in outpatients with major depression. They concluded that, although significant changes in absolute alexithymia levels were observed, alexithymia still evidenced a high degree of relative stability.

There are limitations to this study. First, we relied on participants to provide us with information on self-efficacy, depression, anxiety, and alexithymia, using self-report questionnaires. Future research using alternative methods, such as interviews, might obtain a more complete view of depression and anxiety, self-efficacy, and alexithymia. Second, the sample size was small. The results need to be replicated with a larger group and at multicenter universities. Third, this study is part of “The survey of fourteen psychological factors in Medical students of Babol University of Medical Sciences.” The subjects answered 14 questionnaires as part of a large study. Because of the very numerous questions and questionnaires involved, we had to limit additional measures to assess depression or anxiety symptoms and thus made use of the GHQ-28 subscales. Using the BDI or Beck Anxiety Scale might have led to different results. Fourth, the results reported here likely reflect the influence of cultural factors. The emotional expressing attitude is culturally mediated; functional behavior children learn by imitation from their parents. Finally, as this study is the first on how alexithymia relates to self-efficacy in college students, further research needs to be conducted in order to determine to what extent these factors predict self-efficacy in medical students. In addition, further studies are needed to explain the role of cultural and other variables in mediating the association between alexithymia and self-efficacy.

## 5. Conclusion

This study demonstrate that alexithymia plays significant role in decreasing self-efficacy in academic students. As alexithymia is high in college students and affects self-regulation, goal orientation, and academic function, we suggest that mental physicians routinely evaluate medical college students for alexithymia. In addition, comparative population studies in other countries are needed in order to determine whether risk factors for alexithymia vary among academic students from different cultures. The results of this study have implications for improving self-efficacy and its positive outcomes in universities.

## Figures and Tables

**Table 1:** Demographic characteristics and psychological variable of the sample study.

Age (mean, SD)	21.03 (4.84)
Level of education (*N*, %)	
Doctoral degree	70 (53.6)
Bachelor Science	63 (46.4)
Mothers education (years)	
≤12	85 (63.1)
>12	48 (36.9)
Fathers education (years)	
≤12	65 (48.1)
>12	68 (51.9)
Number of passed semester (*N*, %)	
≤4	70 (53.6)
>4	63 (46.4)
Self-efficacy (mean, SD)	104.72 (23.24)
Depression symptoms (mean, SD)	13.65 (6.37)
Anxiety symptoms (mean, SD)	12.26 (5.37)
Difficulty in identifying feelings (mean, SD)	17.98 (5.06)
Difficulty in describing feelings (mean, SD)	13.39 (3.54)
Externally oriented thinking (mean, SD)	21.41 (3.20)
Total marks of alexithymia (mean, SD)	52.79 (9.02)

*Ranges of scores*. Self-efficacy: 33–165; depression symptoms: 0–28; anxiety symptoms: 0–28; difficulty identifying feelings: 1–35; difficulty describing feelings: 1–25; externally-oriented thinking: 1–40; total alexithymia: 1–100.

**Table 2:** Matrix correlation between depression symptoms, anxiety symptoms, and alexithymia with self-efficacy of academic students.

*Self-efficacy*	1							
*Depression symptoms*	0.086 *P* = 0.354	1						
*Anxiety symptoms*	0.171 *P* = 0.058	0.841 *P* < 0.001	1					
*Difficulty in identifying feelings*	−0.431 *P* < 0.001	−0.099 *P* = 0.272	−0.154 *P* = 0.088	1				
*Difficulty in describing feelings*	−0.397 *P* < 0.001	−0.104 *P* = 0.252	−0.185 *P* = 0.039	0.653 *P* < 0.001	1			
*Externally oriented thinking*	−0.319 *P* < 0.001	0.083 *P* = 0.362	−0.032 *P* = 0.726	0.166 *P* = 0.065	0.179 *P* = 0.046	1		
*Total scores of alexithymia*	−0.0512*P* < 0.001	−0.067 *P* = 0.458	−0.170 *P* = 0.058	0.877 *P* < 0.001	0.823 *P* < 0.001	0.519 *P* < 0.001	1	
	*Self-efficacy*	*Depression symptoms*	*Anxiety symptoms*	*Difficulty in identifying feelings*	*Difficulty in describing feelings*	*Externally oriented thinking*	*Total scores of alexithymia*	1

**Table 3:** The results of multiple analysis of regression (backward) between gender and alexithymia with self-efficacy in academic students.

Model	*B*	SE	Beta	*t*	*P* value

(1) (Constant)	186.122	12.726		14.625	0.000
Gender	−6.880	3.579	−0.152	−1.922	0.057
Alexithymia	−1.342	0.200	−0.530	−6.447	0.000

(2) (Constant)	72.800	10.796		16.006	0.000
Alexithymia	−1.297	0.201	−0.512	−6.447	0.000

Dependent variable: self-efficacy; independent variables: gender and alexithymia.

**Table 4:** The results of multiple analysis of logistic regression (backward) between alexithymia and demographic characteristics of academic students.

Model	*B*	SE	Beta	Wald	*P* value
Step 1					
Educational level (doctoral/bachelor science)	−0.930	0.417	0.395	4.976	0.026
Number of passed semester (≤4/>4)	0.324	0.412	1.383	0.431	0.620
Gender (male/female)	−0.0256	0.404	0.774	0.525	0.431
Mother education (≤12/>12 years)	−0.598	0.506	0.550	0.237	0.237
Father education (≤12/>12 years)	−0.144	0.476	0.866	0.762	0.762
Constant	136.106	82.418	1.289	2.727	0.099

Step 5					
Educational level (doctoral/bachelor science)	−0.657	0.380	0.519	2.292	0.084
Constant	66.371	38.160	6.677	3.025	0.08
